# Clinical impact of volume of disease and time of metastatic disease presentation on patients receiving enzalutamide or abiraterone acetate plus prednisone as first-line therapy for metastatic castration-resistant prostate cancer

**DOI:** 10.1186/s12967-022-03861-2

**Published:** 2023-02-03

**Authors:** Pier Vitale Nuzzo, Filippo Pederzoli, Calogero Saieva, Elisa Zanardi, Giuseppe Fotia, Andrea Malgeri, Sabrina Rossetti, Loana Valenca Bueno, Livia Maria Q. S. Andrade, Anna Patrikidou, Ricardo Pereira Mestre, Mikol Modesti, Sandro Pignata, Giuseppe Procopio, Giuseppe Fornarini, Ugo De Giorgi, Antonio Russo, Edoardo Francini, the SPARTACUSS Investigators

**Affiliations:** 1 Department of Pathology and Laboratory Medicine, Weill Cornell Medicine, New York, NY USA; 2 Cancer Risk Factors and Lifestyle Epidemiology Unit-ISPRO, Florence, Italy; 3 Medical Oncology Unit 1, IRCCS Ospedale Policlinico San Martino, Genoa, Italy; 4 Medical Oncology Department, Fondazione IRCCS Istituto Nazionale Tumori, Milan, Italy; 5 Division of Medical Oncology, Policlinico Universitario Campus Bio-Medico, Rome, Italy; 6 Department of Urology and Gynecology, Istituto Nazionale Tumori IRCCS Fondazione G. Pascale, Naples, Italy; 7 Instituto D’Or de Pesquisa e Ensino, Salvador, State of Bahia Brazil; 8 Hospital São Rafael, Salvador, State of Bahia Brazil; 9 Department of Medical Oncology, Gustave Roussy Institute, Villejuif, France; 10 Istituto Oncologico della Svizzera Italiana, Bellinzona, Switzerland; 11 Oncology Unit, ASST Cremona, Cremona, CR Italy; 12 Department of Medical Oncology, IRCCS Istituto Romagnolo per lo Studio dei Tumori (IRST), Meldola, Italy; 13 Department of Surgical, Oncological, and Oral Sciences, Section of Medical Oncology, University of Palermo, Palermo, Italy; 14 Department of Experimental and Clinical Medicine, University of Florence, Largo Brambilla 3, 50134 Florence, Italy

**Keywords:** Metastatic castration-resistant prostate cancer, Enzalutamide, Abiraterone acetate, Androgen receptor pathway inhibitors, Volume of disease, Metachronous metastases, Synchronous metastases, Prognostic factor

## Abstract

**Background:**

Metastatic castration-resistant prostate cancer remains a challenging condition to treat. Among the available therapeutic options, the androgen receptor signaling inhibitors abiraterone acetate plus prednisone (AA) and enzalutamide (Enza), are currently the most used first-line therapies in clinical practice. However, validated clinical indicators of prognosis in this setting are still lacking. In this study, we aimed to evaluate a prognostic model based on the time of metastatic disease presentation (after prior local therapy [PLT] or de-novo [DN]) and disease burden (low volume [LV] or high-volume [HV]) at AA/Enza onset for mCRPC patients receiving either AA or Enza as first-line.

**Methods:**

A cohort of consecutive patients who started AA or Enza as first-line treatment for mCRPC between January 1st, 2015, and April 1st, 2019 was identified from the clinical and electronic registries of the 9 American and European participating centers. Patients were classified into 4 cohorts by the time of metastatic disease presentation (PLT or DN) and volume of disease (LV or HV; per the E3805 trial, HV was defined as the presence of visceral metastases and/or at least 4 bone metastases of which at least 1 out the axial/pelvic skeleton) at AA/Enza onset. The endpoint was overall survival defined as the time from AA or Enza initiation, respectively, to death from any cause or censored at the last follow-up visit, whichever occurred first.

**Results:**

Of the 417 eligible patients identified, 157 (37.6%) had LV/PLT, 87 (20.9%) LV/DN, 64 (15.3%) HV/PLT, and 109 (26.1%) HV/DN. LV cohorts showed improved median overall survival (59.0 months; 95% CI, 51.0–66.9 months) vs. HV cohorts (27.5 months; 95% CI, 22.8–32.2 months; P = 0.0001), regardless of the time of metastatic presentation. In multivariate analysis, HV cohorts were confirmed associated with worse prognosis compared to those with LV (HV/PLT, HR = 1.87; p = 0.029; HV/DN, HR = 2.19; P = 0.002).

**Conclusion:**

Our analysis suggests that the volume of disease could be a prognostic factor for patients starting AA or Enza as first-line treatment for metastatic castration-resistant prostate cancer, pending prospective clinical trial validation.

## Background

Almost 1.5 million men were diagnosed with prostate cancer and more than 370,000 died of this disease in 2020, worldwide [1]. Metastatic castration-resistant prostate cancer is a lethal state of this disease and typically leads to death in approximately 30 months [2, 3].

The treatment paradigm for metastatic castration-resistant prostate cancer currently encompasses numerous strategies. In real-world clinical practice, the androgen-receptor signaling inhibitors abiraterone acetate plus prednisone (AA) and enzalutamide (Enza) are currently the first and second, respectively, most administered agents as first-line therapy for mCRPC [4]. The biological mechanisms and clinical and genetic factors underlying the efficacy variability of AA or Enza in this setting are still being investigated [5].

A classification based on the time of metastatic disease presentation, whether after prior local therapy (PLT) or de-novo (DN), and volume of disease, whether low volume (LV) or high volume (HV), was previously associated with median overall survival of patients receiving androgen deprivation therapy alone for metastatic castration-sensitive prostate cancer [6–8]. In fact, those with PLT and LV disease had a median overall survival of almost 8 years whereas those with DN and HV had the worst prognosis (about 3.5 years) [6, 7]. Determining whether such a clinical classification is prognostic also for patients starting treatment for metastatic castration-resistant prostate cancer with either second-generation hormone therapy AA or Enza could help clinical counseling as well as optimizing metastatic castration-resistant prostate cancer sequential therapy as previously metastatic castration-sensitive patients progress onto the castration-resistant phase. Therefore, this retrospective analysis aimed to evaluate the prognostic value of a classification model based on the time of metastatic disease presentation and disease burden for patients with metastatic castration-resistant prostate cancer receiving either AA or Enza as first-line treatment in this setting having progressed from a metastatic castration-sensitive state.

## Methods

This cohort study followed the Strengthening the Reporting of Observational Studies in Epidemiology (STROBE) reporting guidelines. Nine institutions in USA, Italy, Switzerland, France, and Brazil participated in this retrospective cohort study. An institutional review board approval was achieved in each center before commencing data collection and a waiver of informed consent was granted owing to all data being de-identified.

A cohort of consecutive patients who started AA or Enza as first-line treatment for histologically confirmed and radiologically evident metastatic castration-resistant prostate cancer between January 1st, 2015, and April 1st, 2019 was identified from the clinical and electronic registries of the 9 participating centers. Those treated with other life-prolonging therapies prior to AA or Enza, apart from androgen deprivation therapy, androgen deprivation therapy plus docetaxel, or androgen deprivation therapy plus radiotherapy, were excluded from the study. The data cut-off date was April 1st, 2022.

Patients were classified into 4 cohorts by the time of metastatic disease presentation (PLT or DN) and volume of disease (LV or HV; per the E3805 trial, HV was defined as the presence of visceral metastases and/or at least 4 bone metastases of which at least 1 out the axial/pelvic skeleton) at AA/Enza onset.

The endpoint was median overall survival, defined as the time from AA or Enza initiation, respectively, to death from any cause or censored at the last follow-up visit, whichever occurred first. Kaplan–Meier method was used to estimate endpoint distributions, including median time-to-event and its 95% confidence interval (CI), while the log-rank test was used to compare time-to-event distributions among the cohorts. The Cox model was applied to assess the time-to-event endpoints in univariate and multivariate models.

## Results

Overall, 417 patients (median age at AA/Enza start, 75 years) were eligible for this analysis and 157 (37.6%) had LV and PLT, 87 (20.9%) LV and DN, 64 (15.3%) HV and PLT, and 109 (26.1%) HV and DN. Median follow-up was 32.5 months (95% CI, 30.6–34.2 months). The main demographic and pathological characteristics of the study population are listed in Table 1.

**Table 1:** Patient characteristics

Variable	Overall series n = 417 No. (%)	LV/PLT n = 157 No. (%)	LV/DN n = 87 No. (%)	HV/PLT n = 64 No. (%)	HV/DN n = 109 No. (%)	p-value*
Median age at start of ARSi, yrs (IQR)	75 (12)	75 (10)	75 (10)	78 (16)	75 (17)	0.14
Race (NA:4)						
Caucasian	373 (90.3)	147 (94.2)	78 (90.7)	59 (92.2)	89 (83.2)	0.24
Black	25 (6.1)	7 (4.5)	4 (4.7)	3 (4.7)	11 (10.3)	
Hispanic	12 (2.9)	2 (1.3)	3 (3.5)	2 (3.1)	5 (4.7)	
Asian	3 (0.7)	0 (0)	1 (1.1)	0 (0)	2 (1.8)	
Gleason score (NA: 53)						
≤ 7	140 (38.5)	77 (54.2)	24 (29.6)	23 (40.4)	16 (19.0)	
8+	224 (61.5)	65 (45.8)	57 (70.4)	34 (59.6)	68 (81.0)	0.0001
Prior local therapy						
No	177 (42.4)	0 (0)	78 (89.7)	0 (0)	99 (90.8)	
Surgery	167 (40.0)	122 (77.7)	7 (8.0)	34 (53.1)	4 (3.7)	
Radiotherapy	73 (17.6)	35 (22.3)	2 (2.3)	30 (46.9)	6 (5.5)	0.0001
Volume at M1 (NA:2)						
Low	291 (70.1)	151 (96.2)	79 (90.8)	29 (45.3)	32 (29.9)	
High	124 (29.9)	6 (3.8)	8 (9.2)	35 (54.7)	75 (70.1)	0.0001
Treatment for mCSPC (NA:47)						
ADT alone	308 (83.2)	120 (97.6)	66 (77.6)	48 (90.6)	74 (67.9)	
ADT + Docetaxel	44 (11.9)	3 (2.4)	6 (7.1)	5 (9.4)	30 (27.5)	
ADT + Radiotherapy	18 (4.9)	0 (0)	13 (15.3)	0 (0)	5 (4.6)	0.0001
ECOG PS at start of ARSi (NA:20)						
0	225 (56.7)	104 (68.0)	56 (66.7)	23 (39.0)	42 (41.6)	0.0001
≥ 1	172 (43.3)	49 (32.0)	28 (33.3)	36 (61.0)	59 (58.4)	
Median PSA at start of ARSi (NA:20)						
ng/mL (IQR)	11.65 (30.52)	7.50 (20.34)	11.90 (28.82)	17.73 (33.78)	21.21 (84.26)	0.001
Treatment discontinuation (NA:2)						
Yes	48 (11.6)	20 (12.8)	11 (12.6)	8 (12.7)	9 (8.3)	0.66
No	367 (88.4)	136 (87.2)	76 (87.4)	55 (87.3)	100 (91.7)	
Number of cycles of 1^st^ line ARSi (NA:47)						
Median (IQR)	13.7 (23.0)	19 (27.6)	19 (23.0)	9 (15.4)	11 (16.0)	0.0001
Median FU in overall population, months (95%CI)	32.5 (30.6–34.2)	38.3	35.1 (31.6–38.6)	24.8 (20.5–28.5)	26.3 (23.2–30.4)	0.0001

*AA* abiraterone acetate plus prednisone, *ADT* androgen deprivation therapy, *CI* confidence interval, *D* docetaxel, *ECOG PS* Eastern Cooperative Oncology Group performance status, *LV* low volume, *HV* high volume, *yrs* years, *IQR* interquartile range, *mCSPC* metastatic castration-sensitive prostate cancer, *PLT* primary local therapy, *DN* de-novo, *NA* not available

^*^p-value from Kruskal–Wallis test or chi-square test, as appropriate

### Overall survival by volume of disease and time of metastatic disease presentation

**Fig. 1:**
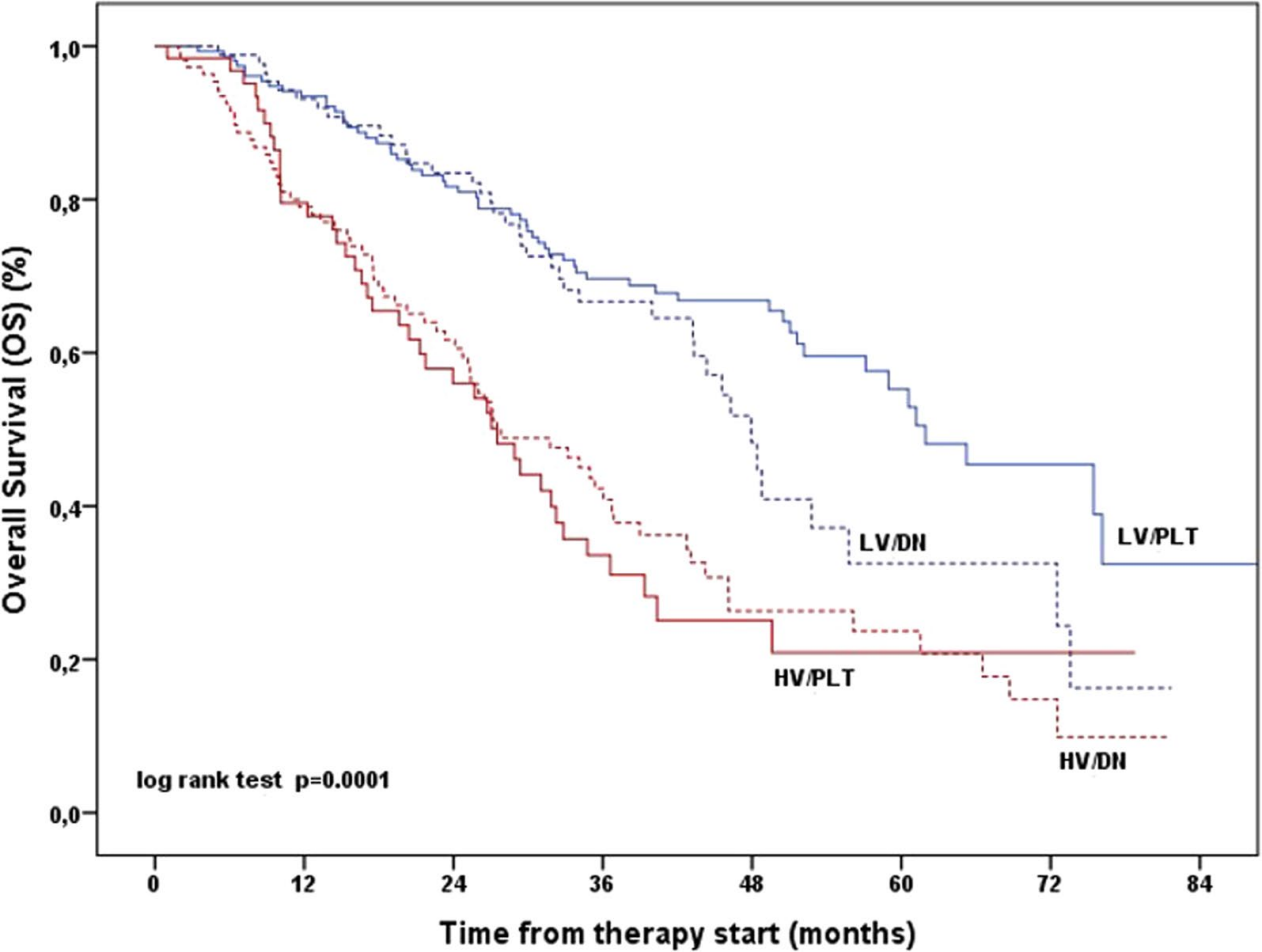
Overall Survival in the overall population by volume of disease at AA/Enza start and time of metastatic disease presentation. *AA* abiraterone acetate plus prednisone, *DN* de-novo, *Enza* enzalutamide, *CI* confidence interval, *HR* hazard ratio, *HV* high volume, *LV* low volume, *OS* overall survival, *PLT* prior local therapy

In univariate analysis, no significant gradient of median overall survival was noted among the 4 cohorts using LV/PLT as a reference (Table 2). However, both cohorts with HV had a greater than double risk of death compared to LV/PLT (HV/DN, Hazard ratio [HR] = 2.63; 95% CI, 1.85–3.74; HV/PLT, HR = 2.84; 95% CI, 1.90–4.26; P = 0.0001). In the Kaplan–Meier survival analysis, the early separation of the curves of the LV cohorts from those of the HV cohorts further highlights these findings (Fig. 1). In multivariate analysis, adjusting for the covariates resulted significant in the univariate model, the cohorts with HV were found independently associated with shortened overall survival compared to those with LV (HV/PLT, HR = 1.90; 95%CI, 1.09–3.32; P = 0.024; HV/DN, HR = 2.17; 95%CI, 1.26–3.73; P = 0.005), along with PSA > 11.4 ng/mL vs. ≤ 11.4 ng/mL (HR = 1.80; 95% CI, 1.27–2.55; P = 0.001) (Table 3).

**Table 2:** Overall survival in the overall population by volume of disease and time of metastatic disease presentation

Cohorts	N. patients (%)	N. deaths (%)	OS (%) (SE)	p-value*	Median OS (months) (95% CI)	HR (95% CI)	p-value°
LV/PLT LV/DN HV/PLT HV/DN	157 (37.6) 87 (20.9) 64 (19.3) 109 (26.2)	59 (28.6) 39 (18.9) 40 (19.4) 68 (33.1)	32.4 (8.9) 16.3 (9.1) 20.9 (6.6) 9.9 (5.4)	0.0001	61.9 (50.0–73.8) 47.9 (43.4–52.5) 27.5 (21.4–33.6) 27.8 (19.8–35.8)	1 (ref) 1.34 (0.90–2.02) 2.84 (1.90–4.26) 2.63 (1.85–3.74)	0.15 0.0001 0.0001

*CI* confidence interval, *DN* de-novo, *HR* hazard ratio, *HV* high volume, *LV* low volume, *OS* overall survival, *PLT* prior local therapy, *SE* standard error

^*^p-value from log-rank test

°p-value from univariate Cox regression analysis

**Table 3:** Multivariate analysis of overall survival in the overall population by volume of disease and time of metastatic disease presentation

	p-value	HR (95% CI)
Subgroup		
LV/PLT		1 (ref.)
LV/DN	0.59	1.15 (0.69–1.94)
HV/PLT	0.024	1.90 (1.09–3.32)
HV/DN	0.005	2.17 (1.26–3.73)
Age		
> 75 *vs* ≤ 75 years	0.20	1.28 (0.88–1.85)
Gleason score		
8 + vs ≤ 7	0.52	1.13 (0.78–1.65)
ECOG PS		
≥ 1 vs 0	0.012	1.66 (1.12–2.45)
Treatment for mCSPC		
ADT alone		1 (ref.)
ADT + D	0.44	0.80 (0.46–1.40)
ADT + Radiotherapy	0.16	0.42 (0.13–1.41)
PSA		
> 11.4 vs ≤ 11.4 ng/mL	0.001	1.80 (1.27–2.55)
Volume at M1		
HV vs LV	0.77	1.07 (0.69–1.65)

*ADT* androgen deprivation therapy, *CI* confidence interval, *D* docetaxel, *DN* de-novo, *ECOG PS* Eastern Cooperative Oncology Group performance status, *HR* hazard ratio, *HV* high volume, *LV* low volume, *M1* time of diagnosis of metastases in the castration-sensitive state, *OS* overall survival, *mCSPC* metastatic castration-sensitive prostate cancer, *PLT* prior local therapy

^*^p-value from log-rank test; °p-value from multivariate Cox regression analysis

### Overall survival by volume of disease

**Fig. 2:**
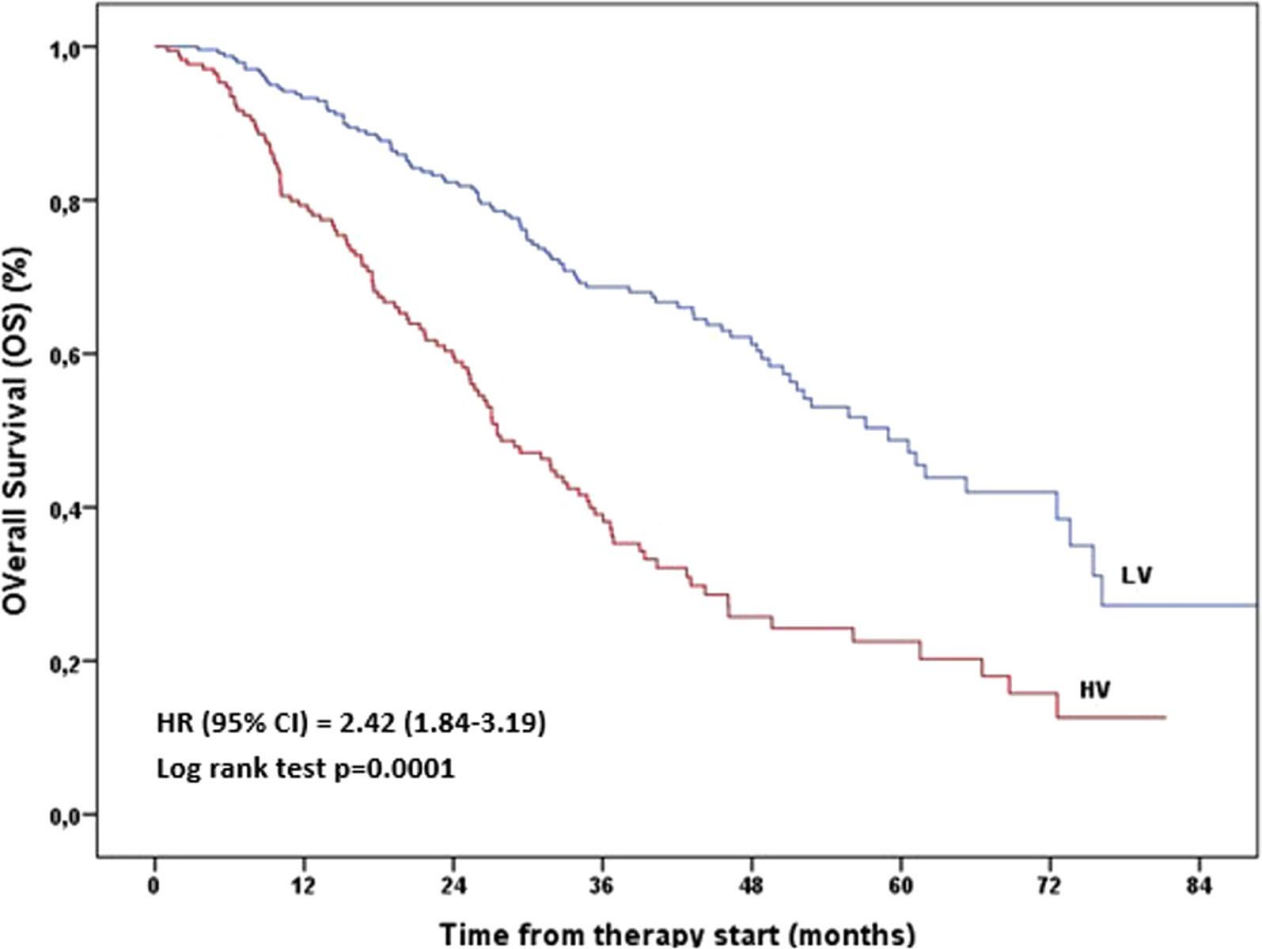
Overall Survival in the overall population by volume of disease at AA/Enza start. AA, abiraterone acetate plus prednisone. *Enza* enzalutamide, *CI* confidence interval, *HR* hazard ratio, *HV* high volume, *LV* low volume, *OS* overall survival

Given the observed lack of survival gradient among the 4 cohorts and the significant prognostic benefit for the cohorts with LV vs those with HV, a secondary analysis was performed to clarify and further detail these findings. Classifying the overall population by the disease burden at AA/Enza onset alone, a significant survival benefit was confirmed for those with LV (59.0 months; 95% CI, 51.0–66.9 months) compared to those with HV (27.5 months; 95% CI, 22.8–32.2 months) and the HV cohort showed a greater than double risk of death than the LV cohort (HR = 2.42; 95% CI, 1.84–3.19; P = 0.0001) (Table 4). The Kaplan–Meier survival curves remark these results describing the early and distinct splitting of the LV and HV curves in favor of the LV cohort (Fig. 2). Interestingly, further classifying LV and HV cohorts according to the time of metastatic disease presentation, no survival difference was found between PLT and DN subgroups in either disease volume cohort (Table 5).

**Table 4:** Overall Survival (OS) in the overall population by volume of disease

Cohorts	N. patients (%)	N. deaths (%)	OS (%) (SE)	p-value*	Median OS (months) (95% CI)	HR (95% CI)	p-value°
LV HV	244 (58.5) 173 (41.5)	98 (47.6) 108 (52.4)	27.2 (6.8) 12.6 (4.5)	0.0001	59.0 (51.0–66.9) 27.5 (22.8–32.2)	1 2.42 (1.84–3.19)	0.0001

*CI* confidence interval, *HR* hazard ratio, *HV* high volume, *LV* low volume, *OS* overall survival, *SE* standard error

^*^p-value from log-rank test; °p-value from univariate Cox regression analysis

**Table 5:** Overall survival in the LV and HV cohorts according to time of metastatic disease presentation

LV cohort (n = 244)	N. patients (%)	N. deaths (%)	OS (%) (SE)	p-value*	Median OS, months (95% CI)	HR (95% CI)	p-value°
PLT DN	157 (64.3) 87 (35.7)	59 (60.2) 39 (39.8)	32.4 (8.9) 16.3 (9.1)	0.11	61.9 (50.0–73.8) 47.9 (43.4–52.5)	1 (ref) 1.39 (0.93–2.10)	0.11

HV cohort (n = 173)	N. patients (%)	N. deaths (%)	OS (%) (SE)	p-value*	Median OS months (95% CI)	HR (95% CI)	p-value°
PLT DN	64 (37.0) 109 (63.0)	40 (37.0) 68 (63.0)	20.9 (6.6) 9.9 (5.4)	0.71	27.5 (21.4–33.6) 27.8 (19.8–35.8)	1 (ref) 0.93 (0.63–1.37)	0.71

*CI* confidence interval, *DN* de-novo, *HR* hazard ratio, *HV* high volume, *LV* low volume, *OS* overall survival, *PLT* prior local therapy, *SE* standard error

^*^p-value from log-rank test; °p-value from univariate Cox regression analysis

## Discussion

Prior studies demonstrated the prognostic value of a clinical model based on the time of metastatic disease presentation and disease burden for patients with metastatic castration-sensitive prostate cancer receiving androgen deprivation therapy alone [6–8]. This retrospective multicenter analysis shows that the volume of disease, evaluated at the onset of first-line AA/Enza for metastatic castration-resistant prostate cancer, remains a valid prognostic factor for patients receiving either AA or Enza for first-line metastatic castration-resistant prostate cancer, regardless of whether PLT or DN. In fact, patients with HV had a median overall survival more than halved compared to that of LV (59.0 vs. 27.5 months) and their risk of death was more than two-fold that of men with LV (HR = 2.42). Prospective randomized data on the impact of disease burden on the survival of patients with metastatic castration-resistant prostate cancer is currently scarce in the literature. However, pivotal phase 3 trials of AA or Enza as first-line therapy for metastatic castration-resistant prostate cancer included patients with asymptomatic or mildly symptomatic disease spread to the nodes and/or bones only and pre-treated with androgen deprivation therapy alone for metastatic castration-sensitive prostate cancer, that is a population likely with LV [9]. Although indirectly, this observation supports our findings on LV disease being associated with prolonged survival when patients are treated with AA/Enza as first-line. Conversely, the time of metastatic disease presentation showed no association with survival in this cohort study. In this respect, as opposed to what was observed in the metastatic castration-sensitive setting [6, 7], HV/DN did not show the worst prognosis among the 4 cohorts. Notably, almost 30% of men with DN and HV at prostate cancer diagnosis had LV when radiographically re-evaluated at AA/Enza onset whereas the vast majority of those with DN and LV at diagnosis (90.8%) stayed LV at the start of AA/Enza. This disease burden “migration” observed in those with DN and HV at diagnosis, maybe due to the success of therapy used for metastatic castration-sensitive prostate cancer, could partly explain the lack of impact of time of metastatic disease presentation on the survival of patients in this setting. Over the past decade, the treatment armamentarium for metastatic castration-resistant prostate cancer has grown larger, and optimizing therapy sequencing has become an unmet clinical need [10].

## Conclusion

Albeit limited by a retrospective design and restricted sample size, this international multicenter analysis suggests that the volume of disease could be a prognostic factor for metastatic castration-resistant prostate cancer patients starting AA or Enza as first-line. If validated in large prospective clinical trials, the disease burden could inform the design of future randomized studies in this setting and ultimately provide a simple clinical tool to aid treatment-decision making.

## Data Availability

The datasets used and/or analysed during the current study are available from the corresponding author on reasonable request. Edoardo Francini had full access to all the data in the study and takes responsibility for the integrity of the data and the accuracy of the data analysis.

## Abbreviations

AA: Abiraterone acetate plus prednisone
CI: Confidence interval
DN: De-novo
ECOG PS: Eastern Cooperative Oncology Group performance status
Enza: Enzalutamide
HR: Hazard ratio
HV: High-volume
LV: Low volume
PLT: Prior local therapy
STROBE: Strengthening the Reporting of Observational Studies in Epidemiology
